# Protocol for a parallel cluster randomized trial of a participatory tailored approach to reduce overuse of antibiotics at hospital discharge: the ROAD home trial

**DOI:** 10.1186/s13012-024-01348-w

**Published:** 2024-03-04

**Authors:** Julia E. Szymczak, Lindsay A. Petty, Tejal N. Gandhi, Robert A. Neetz, Adam Hersh, Angela P. Presson, Peter K. Lindenauer, Steven J. Bernstein, Brandi M. Muller, Andrea T. White, Jennifer K. Horowitz, Scott A. Flanders, Justin D. Smith, Valerie M. Vaughn

**Affiliations:** 1 Department of Internal Medicine, Division of Epidemiology, University of Utah School of Medicine, 295 Chipeta Way, Salt Lake City, UT 84132 USA; 2 Department of Internal Medicine, Division of Infectious Diseases, Michigan Medicine, University of Michigan, Ann Arbor, MI USA; 3 MyMichigan Medical Center Midland, MyMichigan Health, Midland, MI USA; 4 Department of Pediatrics, Division of Pediatric Infectious Diseases, School of Medicine, University of Utah, Salt Lake City, UT USA; 5 Baystate Medical Center Department of Healthcare Delivery and Population Science, Center for Quality of Care Research, Springfield, MA USA; 6 Medicine Service, Veterans Affairs Ann Arbor Healthcare System, Ann Arbor, MI USA; 7 Department of Internal Medicine, Division of General Internal Medicine, Michigan Medicine, University of Michigan, Ann Arbor, MI USA; 8 Center for Clinical Management Research, Veterans Affairs Ann Arbor Healthcare System, Ann Arbor, MI USA; 9 Department of Internal Medicine, Division of General Internal Medicine, University of Utah School of Medicine, 295 Chipeta Way, Salt Lake City, UT 84132 USA; 10 Department of Internal Medicine, Division of Hospital Medicine, Michigan Medicine, University of Michigan, Ann Arbor, MI USA; 11 Department of Population Health Sciences, Division of Health System Innovation & Research, University of Utah School of Medicine, Salt Lake City, USA

**Keywords:** Antibiotic stewardship, Hospital discharge, Urinary tract infection, Pneumonia, Facilitation, Tailoring, Integrated Promoting Action on Research Implementation in Health Services Research (i-PARIHS) framework, Transitions of care

## Abstract

**Background:**

Antibiotic overuse at hospital discharge is common, costly, and harmful. While discharge-specific antibiotic stewardship interventions are effective, they are resource-intensive and often infeasible for hospitals with resource constraints. This weakness impacts generalizability of stewardship interventions and has health equity implications as not all patients have access to the benefits of stewardship based on where they receive care. There may be different pathways to improve discharge antibiotic prescribing that vary widely in feasibility. Supporting hospitals in selecting interventions tailored to their context may be an effective approach to feasibly reduce antibiotic overuse at discharge across diverse hospitals. The objective of this study is to evaluate the effectiveness of the Reducing Overuse of Antibiotics at Discharge Home multicomponent implementation strategy (“ROAD Home”) on antibiotic overuse at discharge for community-acquired pneumonia and urinary tract infection.

**Methods:**

This 4-year two-arm parallel cluster-randomized trial will include three phases: baseline (23 months), intervention (12 months), and postintervention (12 months). Forty hospitals recruited from the Michigan Hospital Medicine Safety Consortium will undergo covariate-constrained randomization with half randomized to the ROAD Home implementation strategy and half to a “stewardship as usual” control. ROAD Home is informed by the integrated-Promoting Action on Research Implementation in Health Services Framework and includes (1) a baseline needs assessment to create a tailored suite of potential stewardship interventions, (2) supported decision-making in selecting interventions to implement, and (3) external facilitation following an implementation blueprint. The primary outcome is baseline-adjusted days of antibiotic overuse at discharge. Secondary outcomes include 30-day patient outcomes and antibiotic-associated adverse events. A mixed-methods concurrent process evaluation will identify contextual factors influencing the implementation of tailored interventions, and assess implementation outcomes including acceptability, feasibility, fidelity, and sustainment.

**Discussion:**

Reducing antibiotic overuse at discharge across hospitals with varied resources requires tailoring of interventions. This trial will assess whether a multicomponent implementation strategy that supports hospitals in selecting evidence-based stewardship interventions tailored to local context leads to reduced overuse of antibiotics at discharge. Knowledge gained during this study could inform future efforts to implement stewardship in diverse hospitals and promote equity in access to the benefits of quality improvement initiatives.

**Trial registration:**

Clinicaltrials.gov NCT06106204 on 10/30/23

**Supplementary Information:**

The online version contains supplementary material available at 10.1186/s13012-024-01348-w.

Contributions to the literature

- This 40-hospital randomized controlled trial will test the effectiveness of a multicomponent implementation strategy to reduce the overuse of antibiotics at hospital discharge, which is common, costly, and harmful.
- The strategy is designed to promote equity in antibiotic stewardship by encouraging fit between interventions and hospital context so the benefits of stewardship can reach patients wherever they receive care.
- This trial will advance implementation science by improving understanding of the way tailoring, a commonly used but ill-defined strategy, works through precise specification of timing, people involved, steps in the process, and potential mechanisms of action.

## Introduction

Antibiotic overuse is common, costly, and harmful [1–3]. Antibiotics prescribed at hospital discharge account for half of antibiotic days received by hospitalized adults with infections in the United States (US) [4–9]. Yet, up to 70% of discharge antibiotic prescriptions are not guideline concordant; rather, they are unnecessary, have an excessive duration, or use a suboptimal agent [4, 5, 8]. Community-acquired pneumonia (CAP) and urinary tract infection (UTI), which together account for half of all hospital-related antibiotic use [10], are particularly prone to antibiotic overuse at discharge [11]. Antibiotic overuse at hospital discharge increases antibiotic-associated adverse events and antibiotic resistance, especially among vulnerable patients such as those residing in long-term care facilities [12–14].

While some stewardship interventions to improve discharge antibiotic use have proved efficacious [15–19], data are limited on how to implement them across hospitals with varied resources and capacity [20]. For example, stewardship interventions such as infectious diseases (ID)-trained pharmacist-led audit and feedback or fluoroquinolone restriction integrated into hospital discharge planning have reduced antibiotic overuse at discharge [15]. However, these approaches are resource-intensive and often infeasible or unsustainable at hospitals with limited resources (e.g., those without ID pharmacists) or shifting priorities. This limitation impacts the generalizability of successful stewardship interventions; though nearly all US hospitals have antimicrobial stewardship programs (ASPs), they vary considerably in their capacity to implement evidence-based but resource-intensive interventions [21, 22].

Insufficient knowledge of how to improve discharge antibiotic use across variable contexts may contribute to health inequity, as not all patients have access to the benefits of robust ASPs based on where they receive care [23]. Thus, there is an urgent need to design stewardship interventions with broad dissemination in mind [24, 25]. Too often, investigators do not consider the fit between an intervention and the places it could be implemented [26, 27]. It is well established that “one size doesn’t fit all” in stewardship [28–30], but existing research does not adequately specify the mechanisms by which the tailoring of stewardship interventions to context occurs [31], a process that is ill-defined in implementation science more generally [32]. Poor specification of these tailoring mechanisms creates difficulties for antibiotic stewardship research by hampering the interpretation of results, replication of intervention studies, and evidence synthesis. It also impedes scale up and broad implementation of stewardship interventions found to be effective in one setting to others.

Evidence-based antibiotic stewardship interventions often involve individual or combinations of discrete strategies (e.g., prospective audit and feedback, updated guidelines, clinician education, behavioral nudges, clinical decision support) to move evidence-based prescribing practices (guideline-concordant antibiotic use—the right drug for the right diagnosis, at the right dose, for the right duration) [33] into routine clinical practice by changing clinician behavior [34].^1^ In the case of discharge antibiotic stewardship interventions, emerging evidence suggests there are different pathways to improve antibiotic use that vary widely in feasibility [35]. Supporting hospitals in selecting evidence-based stewardship interventions tailored to their local context is likely more feasible and effective for diverse hospitals than a “one size fits all” approach to antibiotic stewardship at discharge which suggests, for example, that all hospitals should implement an ID pharmacist-led audit and feedback program [28]. Little is known about whether or how tailoring works in antibiotic stewardship to improve prescribing.

In this trial, we will be investigating the impact of a multicomponent implementation strategy (referred to as “the ROAD Home strategy”) on discharge antibiotic prescribing for hospitalized adults with CAP and UTI. The ROAD Home strategy is intended to help hospitals select, tailor, and implement evidence-based interventions to improve discharge antibiotic prescribing (referred to as “stewardship interventions”).

### Hypothesis and aims

The Reducing Overuse of Antibiotics at Discharge (ROAD) Home study is designed to test the hypothesis that hospitals randomized to a tailored, multicomponent implementation strategy that includes external facilitation while allowing for local autonomy in selecting and implementing evidence-based stewardship interventions will have fewer days of antibiotic overuse at discharge than “stewardship as usual” in control hospitals.

The study has two aims: (1) to evaluate the effectiveness of the ROAD Home strategy on days of antibiotic overuse at discharge for hospitalized patients treated for CAP and UTI; and (2) to identify contextual factors influencing tailoring and implementation of tailored interventions, and assess implementation outcomes (acceptability, feasibility, fidelity, and sustainment).

## Methods and analysis

### Trial design

A two-arm, parallel, cluster-randomized trial will assess the effect of the ROAD Home strategy on days of antibiotic overuse at discharge. We will recruit at least 40 hospitals from within the Michigan Hospital Medicine Safety Consortium (HMS), a statewide 69-hospital collaborative consisting of diverse hospitals focused on improving the care of hospitalized patients. HMS hospitals that agree to participate will undergo covariate-constrained randomization to improve balancing of hospital characteristics between groups with 1:1 allocation to the ROAD Home strategy vs. a “usual stewardship” control. In the 12-month intervention period, hospitals will implement their selected stewardship interventions while we assess days of antibiotic overuse at discharge and patient outcomes. During the intervention period and in the 12-month postintervention period, we will conduct a mixed-methods process evaluation to evaluate barriers, facilitators, and implementation outcomes across hospitals.

### Study setting

HMS is supported by Blue Cross/Blue Shield of Michigan (BCBSM) and, since 2017, has focused on improving the care of hospitalized patients treated for UTI or CAP. Trained abstractors at each hospital collect data via medical record review of a pseudo-random sample of hospitalized patients with a positive urine culture or CAP within each hospital [7, 36, 37]. There are tri-annual HMS meetings (2 in person, 1 virtual) to share data and best practices. HMS was initially voluntary (pre-2020) but has become required for all eligible hospitals participating in BCBSM’s value-based partnership program. HMS has established pay for performance antibiotic use metrics which promote improvement. However, HMS does not provide financial support for antibiotic stewardship interventions nor specify how hospitals should achieve improvement. To date, there remains variation in performance on antibiotic performance metrics across HMS hospitals [36, 37]. Recruitment from HMS is highly pragmatic due to existing infrastructure which allows patient-level data collection, hospital survey administration, in person group meetings, and inclusion of diverse hospitals from across the state of Michigan. As of January 2023, 69 (75%) of the 92 non-critical access, non-federal hospitals in the state of Michigan participate in HMS. Table 1 shows characteristics of HMS hospitals.

**Table 1:** HMS hospital characteristics, *n* = 69 hospitals

Bed size; median (IQR)	277 (169–381.5)
Rurality; *n* (%)	
Metropolitan (RUCC 1–3)	55 (80%)
Rural	14 (20%)
Somewhat rural (RUCC 4–6)	7 (10%)
Very rural (RUCC 7–9)	7 (10%)
Leader of antibiotic stewardship program; *n* (%)^a^	
ID physician and ID pharmacist	30 (43%)
Other	39 (56%)
ID physician OR ID pharmacist	34 (49%)
Neither ID-trained	5 (7%)
ID availability; *n* (%)^a^	
Onsite daily	45 (65%)
Onsite occasionally	10 (14%)
Remote only (phone or virtual)	8 (12%)
None	6 (9%)
Minority serving populations; median (IQR)	18.1% (8.7%–35.6%)
Safety net populations; median (IQR)	8.1% (5.3%–11.7%)
Baseline number of ROAD Home interventions; median (IQR)^a,b^	
Unweighted	15 (11–18)
Weighted	27 (20–32)
Baseline number of discharge-specific interventions; *n* (%)^a^	
0	35 (51%)
1	25 36%)
≥ 2	9 (13%)
Part of system; *n* (%)	
National	16 (23%)
State	48 (70%)
No	5 (7%)
Electronic medical record vendor	
Epic	35 (51%)
Cerner	21 (30%)
Other	12 (17%)
Not reported	1 (1%)

*HMS* Michigan Hospital Medicine Safety Consortium, *RUCC* Rural–urban continuum code, *ID* Infectious diseases, *IQR* Interquartile range, *ROAD* Reducing overuse of antibiotics at hospital discharge

^a^Data element ascertained through HMS annual survey

^b^Weighting refers to the point value given to each intervention in use (each Tier 1 intervention = 1 point, each Tier 2 intervention = 2 points, each Tier 3 intervention = 3 points)

### Participants

Stakeholders working in acute care hospitals who implement stewardship interventions (inner setting implementation leads) are the primary targets of the strategy being evaluated in the ROAD Home trial. These inner setting implementation leads will be the main point of contact between the ROAD Home study team and hospitals randomized to receive the ROAD Home strategy. Each hospital within HMS is represented by at least one data abstractor (usually a nurse with quality improvement training) and one physician champion. Many hospitals also include quality improvement representatives or leaders, administrators, additional physicians, and pharmacists involved in antibiotic stewardship. Given the variation in stewardship stakeholder composition that naturally exists in HMS, we will allow hospitals to self-select the individual(s) to represent them as implementation lead(s) within the trial.

Because the ROAD Home strategy is intended to increase the uptake of antibiotic stewardship interventions known to reduce antibiotic overuse at hospital discharge, patients cared for in intervention arm hospitals who are being treated for UTI or CAP are secondary targets, in that increased uptake of evidence-based antibiotic prescribing is expected to translate into improved clinical outcomes.

### Inclusion/exclusion criteria

All hospitals participating in HMS are eligible to participate.

### Recruitment

Hospital recruitment occurred in November 2023 at an in-person HMS collaborative meeting. Hospitals unable to attend the in-person meeting were contacted for a separate, virtual meeting in December 2023. Representatives from eligible hospitals were invited to an in-person recruitment session led by the ROAD Home investigative team. To improve trial retention and commitment to study procedures, we employed an adapted Methods Motivational Interviewing approach [38] for recruitment where we (a) set clear participant expectations, (b) introduced the trial including rationale for key elements of study design, and (c) diffused ambivalence about trial participation through small group discussion. This process has been shown to improve study retention in behavioral clinical trials [39]. Recruitment is still ongoing. The recruitment goal, based on power calculations (see below), was at least 40 hospitals; given the goals of HMS, we did not turn away hospitals interested in participating once our goal of 40 was reached. As of December 20, 2023, 50 hospitals have agreed to participate.

### Randomization

**Fig. 1:**
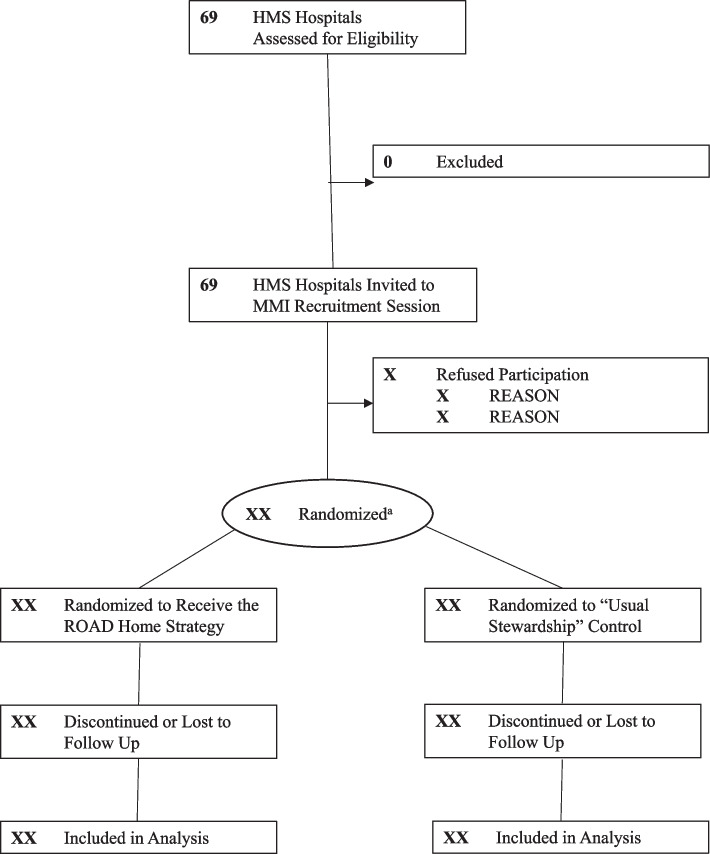
CONSORT diagram. HMS, Michigan Hospital Medicine Safety Consortium; MMI, methods motivational interviewing. ^a^Hospitals will undergo covariate-constrained randomization to improve balancing of critical characteristics between groups with 1:1 allocation

**Fig. 2:**
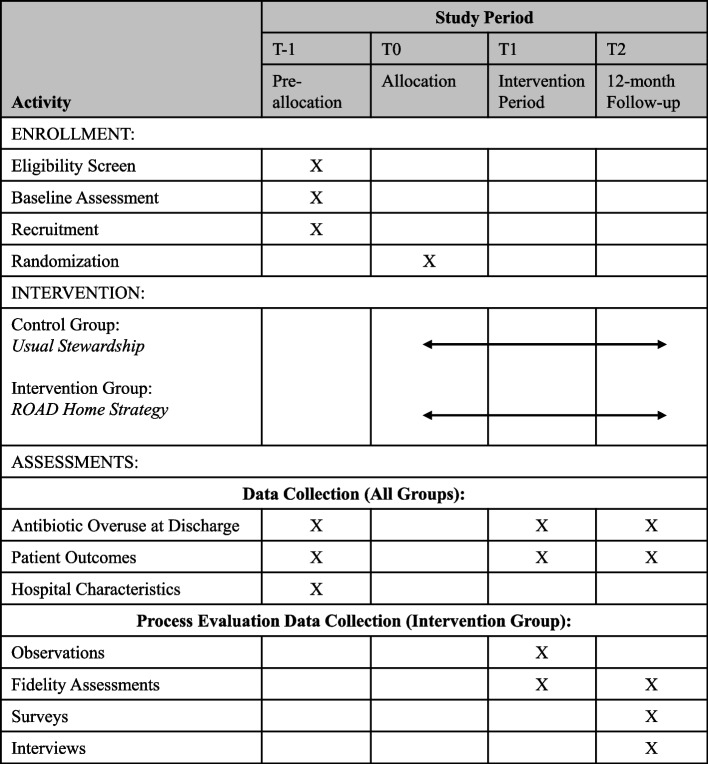
SPIRIT flow diagram: schedule of enrollment, interventions, and assessments

The trial will randomize study hospitals in a 1:1 allocation ratio to two arms: “ROAD Home strategy” intervention vs. “usual stewardship” control (see Fig. 1 for CONSORT Diagram). Eligible hospitals that agree to participate will undergo covariate-constrained randomization to improve balancing of critical hospital characteristics between groups. Covariate-constrained randomization allows for balancing of multiple pre-specified characteristics of interest when the number of characteristics is high compared to the number of clusters being randomized [40]. Characteristics that will be included in the constrained randomization process include (a) baseline antibiotic overuse at discharge; (b) year of entry into HMS; (c) social needs of population being served by the hospital (classified as rural hospital) (defined as either somewhat or very rural using Rural Urban Continuum Code (RUCC) score > 3) [41], Minority Serving Hospital or Safety Net Hospital (defined as hospital with RUCC score of 1 or 2 and either top 25% non-white and/or Hispanic population or top 25% Medicaid or uninsured population, respectively) [42] or neither; (d) composition of ASP leadership (is the ASP team led by both an ID physician and an ID pharmacist or not); and (e) baseline number of stewardship interventions already implemented (weighted using our published ROAD Home framework) [35]. We will select from randomization sequences that have a balance of ± 10% or ± 2 sites between arms for continuous and categorical variables, respectively. All hospitals will be allocated to intervention or control arms simultaneously, negating the need for allocation concealment (see Fig. 2 for SPIRIT flow diagram). Due to the need for investigators to participate in the delivery of the ROAD Home strategy, neither investigators nor hospitals will be blinded to their assignment.

### Intervention hospitals

#### Development of ROAD Home

ROAD Home is a multicomponent implementation strategy that combines (a) evaluative techniques to understand hospital context, (b) tailoring of stewardship interventions to that context, (c) external facilitation, and (d) active participation from hospitals to address known barriers to the implementation of discharge antibiotic stewardship. We developed ROAD Home based on extensive formative research on the current discharge stewardship landscape, approaches to reduce discharge overuse across hospital contexts, and quantitative associations of different stewardship interventions with antibiotic overuse at discharge [7, 35, 43, 44]. This research demonstrated three primary findings that informed the creation of ROAD Home. First, the stewardship intervention that has demonstrated efficacy and is most recommended (prospective audit and feedback on discharge antibiotic prescriptions by a clinical or ID pharmacist) [15] to improve discharge prescribing is resource intensive and not feasible or sustainable for most hospitals, and thus rarely used [7, 16, 44–47]. Second, there appear to be multiple pathways to improve discharge antibiotic prescribing through combinations of stewardship interventions that vary widely in feasibility [4, 35]. Third, antibiotic stewardship interventions that are implemented using a participatory approach are more likely to be successful than “top down” approaches because they promote stakeholder engagement and tailoring of strategies to context [30, 48–53].

**Fig. 3:**
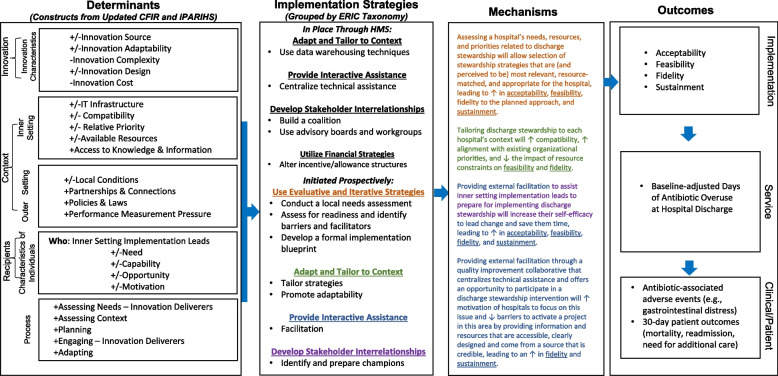
ROAD Home Trial Implementation Research Logic Model. CFIR, Consolidated Framework for Implementation Research, Damschroder et al. *Implementation Science* (2022). ERIC, Expert Recommendations for Implementing Change, Powell et al. *Implementation Science* (2015). HMS, Michigan Hospital Medicine Safety Consortium. Implementation Research Logic Model Template from Smith et al. *Implementation Science* (2020). Color coding indicates the connection between the ERIC strategy and mechanism proposed for how it impacts implementation outcomes (underlined). +  = facilitator.—= barrier

Considering these findings in concert with the integrated-Promoting Action on Research Implementation in Health Services (iPARIHS) framework, we selected a group of implementation strategies that are likely to promote the tailoring of stewardship interventions to improve discharge antibiotic prescribing [54]. iPARIHS suggests that successful implementation is a function of the quality of the *evidence* to be implemented and the way the stakeholders perceive this evidence; the characteristics of the setting or *context* in which implementation occurs; and the way that evidence is introduced or *facilitated* into practice [55]. Facilitation is central in iPARIHS, which posits that facilitation enables successful implementation by connecting action around new practices with the people who need to implement the new practice in a context-adaptive manner [56]. ROAD Home is intended to support inner setting implementation leads tasked with quality improvement and/or antibiotic stewardship to implement interventions to improve antibiotic use at hospital discharge. An additional file includes a table that specifies each strategy included in ROAD Home (see Additional file 1) [57]. Figure 3 depicts our Implementation Research Logic Model [58] elaborating the connections between implementation determinants, strategies, and outcomes addressed in the ROAD Home Trial.

### The ROAD Home strategy

**Fig. 4:**
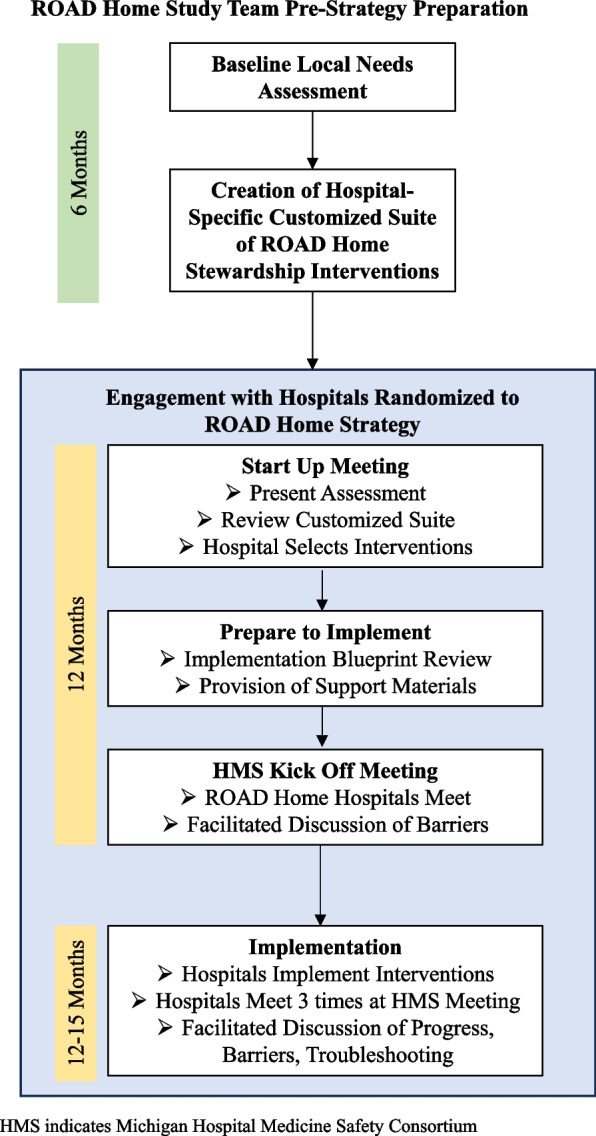
ROAD Home strategy. HMS, Michigan Hospital Medicine Safety Consortium

Hospitals randomized to receive ROAD Home will undergo (1) a baseline needs assessment to create a tailored suite of discharge stewardship interventions, (2) supported decision-making in selecting interventions to implement, and (3) external facilitation following an implementation blueprint (Fig. 4). We describe each strategy below.

#### Baseline needs assessment and development of customized suite of stewardship interventions

Hospitals will undergo a baseline assessment including an audit of baseline antibiotic use at discharge to identify areas of high overuse and a needs assessment. The needs assessment—conducted via HMS annual survey—will assess antibiotic stewardship interventions currently in use (and, if in use, how frequently, on what patient populations, and by whom), existing resources, infrastructure, hospital priorities, and anticipated barriers to implementation. Based on the findings of this local needs assessment, study investigators will generate a tailored suite of potential stewardship interventions for each hospital.

The tailored suite created for each hospital randomized to receive ROAD Home is based on a framework we developed that specifies a three-tiered suite of evidence-based discharge antibiotic stewardship interventions [4, 11, 35]. Tier 1 includes critical stewardship infrastructure (e.g., guidelines); Tier 2 includes inpatient-focused stewardship interventions (e.g., inpatient-focused audit and feedback); and Tier 3 includes discharge-focused stewardship interventions (e.g., audit and feedback of discharge prescriptions). While Tier 3 interventions individually have been shown to be most efficacious [15, 17, 19, 35], we found in a retrospective analysis [35] that implementing three Tier 1 interventions, or one Tier 1 and one Tier 2 intervention, had a similar effect on antibiotic prescribing at discharge as implementing a single Tier 3 intervention. Thus, rather than suggesting all hospitals implement the same intervention to improve discharge prescribing, ROAD Home supports hospitals in selecting intervention(s) to implement based on their existing priorities, resources, and infrastructure [35]. Interventions have been given a point value (each Tier 1 intervention = 1 point, each Tier 2 intervention = 2 points, each Tier 3 intervention = 3 points). The only requirement is that hospitals select 3 points worth of interventions to implement, which they can achieve by selecting a single Tier 3 intervention, or a combination of Tier 2 and Tier 1 interventions, or three Tier 1 interventions.

**Fig. 5:**
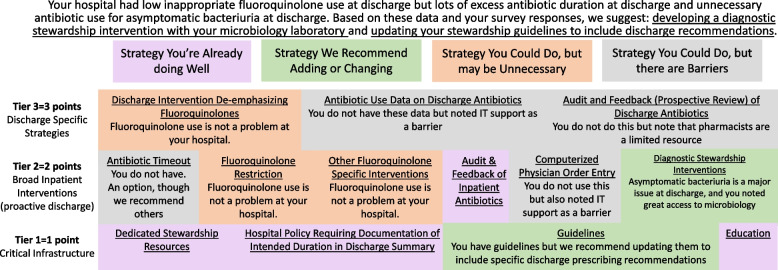
Example tailored ROAD Home suite

Each hospital will receive a tailored ROAD Home suite with a personalized graphic (see Fig. 5) that specifies ROAD Home interventions at each tier placed in one of four groups: (a) already doing well (no need to change); (b) intervention we recommend adding/changing; (c) intervention your hospital could implement, but may be unnecessary given performance; or (d) intervention your hospital could implement, but there are barriers. Recommended interventions will add up to 3 points but can be changed based on hospital preference.

#### Supported decision-making to select and tailor interventions

After intervention hospitals review their tailored suite, they will meet virtually with study investigators to ask questions, provide clarifying information, and suggest edits to which 3 points they choose from the suite. Stewardship interventions not included in the original suite may also be considered; novel interventions will be given a point value determined by study investigators based on the most appropriate Tier fit. With assistance from the study investigators, intervention hospital implementation lead(s) will select a total of 3 points of interventions to implement. While study investigators will facilitate a discussion of the anticipated benefits, drawbacks, barriers, facilitators, and resources needed for each potential intervention, the hospital implementation lead(s) will make the final decision on which interventions to include.

#### External facilitation following an implementation blueprint

Once interventions have been selected, study investigators will facilitate a discussion about implementation, starting with the hospital implementation lead(s) selecting a start date within a 3–6-month window and creating an implementation blueprint. The blueprint will map out the specific steps, a timeframe with milestones, responsible people (or groups), and resources needed to implement ROAD Home interventions. An additional file shows a sample implementation blueprint (see Additional file 2) [59]. The hospital implementation lead(s) will be provided with a blueprint template, instructions to fill it out, and a request to submit it for review within 1 month. Study investigators will review the blueprint for completeness and ask clarifying questions if necessary. As intervention hospitals prepare to implement their selected interventions, they will have access to adaptable tools from study investigators to support implementation of the selected interventions. For example, if a hospital decides (for 1 point) to create and implement institution specific UTI guidelines, we will provide a guideline template that can be tailored (e.g., adding their hospital logo, utilizing context-specific language).

As part of external facilitation, all hospitals will have 4 additional meetings with study investigators and other intervention hospitals during HMS meetings. The first, just prior to the intervention period, will be a “kick-off” meeting where study investigators will facilitate a discussion among hospital implementation leads including a high-level presentation of themes that cut across the implementation blueprints related to anticipated barriers. The purpose of this meeting is to engage meaningfully with intervention hospitals prior to launch, build enthusiasm for the approach, and develop a sense of camaraderie across sites. During the intervention period, hospitals will have 2 “check-in” sessions and 1 close out session during HMS collaborative wide meetings. These meetings will be led by the study investigators who will facilitate a discussion about study progress, challenges, and strategies to overcome challenges. These meetings have three primary functions to facilitate implementation: (1) foster a learning climate among hospitals so that implementation leads can share with each other strategies they have used to address barriers; (2) promote accountability for follow through on the planned ROAD Home interventions, which will increase fidelity to the implementation blueprints; and (3) increase implementation leads’ self-efficacy to make change at their hospital.

### Control hospitals

The study’s primary aim is to understand whether the ROAD Home strategy results in fewer days of antibiotic overuse at discharge compared to “usual stewardship.” With this goal in mind, hospitals randomized to the control group will continue usual HMS activities and their existing stewardship interventions. As described previously [36, 37], usual HMS activities include hospital benchmarking of key quality metrics (e.g., pneumonia antibiotic duration), sharing best practices during 3 times per year meetings, and pay-for-performance metrics. These activities may touch on discharge prescribing (e.g., during best practices discussion) but discharge prescribing will not be the focus (i.e., pay-for-performance metrics will not be discharge antibiotic use). Individual hospital activities are not specific to HMS but hospitals often use HMS data to improve antibiotic prescribing. It is possible that some control hospitals may decide to focus on improving discharge antibiotic use during the intervention period. However, they will not receive any of the ROAD Home strategies including baseline data analysis or needs assessment, tailored suite, supported decision-making in selecting interventions to implement, an implementation blueprint, adaptable stewardship tools, or external facilitation. We will assess ongoing and new stewardship interventions (including discharge-focused interventions) implemented in both intervention and control hospitals via annual required HMS surveys to identify which, if any, control hospitals worked on improving discharge antibiotic use during the intervention period and conduct a per protocol sensitivity analysis that excludes control hospitals that started a new discharge-focused stewardship intervention.

### Study outcomes

#### Primary outcome

Our primary study outcome will be baseline-adjusted days of antibiotic overuse at discharge, defined by our extensively tested, guideline-based method (with minor updates applied to reflect national guideline changes) [4, 11, 35]. Antibiotic overuse at discharge includes a composite of unnecessary antibiotic use (e.g., antibiotics for asymptomatic bacteriuria [ASB]), excessive antibiotic duration (e.g., > 3–5 days for most patients with CAP), and avoidable fluoroquinolones (e.g., ciprofloxacin use for a patient with uncomplicated cystitis who has no allergies).

#### Balancing metric

Prior data suggest that excessive antibiotic use does not improve patient outcomes [60]. To ensure no unintended patient harm occurs from reducing antibiotic overuse at discharge, we will use a non-inferiority framework to analyze composite 30-day post-discharge patient outcomes including mortality, readmission, or urgent visit (i.e., urgent care or emergency care visit).

#### Secondary outcome

Our composite clinical secondary outcome is antibiotic-associated adverse events defined using our prior metrics [4, 7, 61] including common side effects such as gastrointestinal upset and candidiasis as well as potentially life-threatening side-effects such as *Clostridioides difficile* infection (CDI). Side effects will be gathered through a combination of chart review and 30-day post-discharge patient phone calls.

### Data collection, completeness, and quality

Antibiotic overuse at discharge—including the clinical data needed to define “overuse”—will be collected as part of HMS’ ongoing quality improvement initiatives. In HMS, discharge antibiotic prescriptions and discharge summaries are reviewed by trained clinical abstractors who collect antibiotic name, start, and stop dates, discharge antibiotic duration, number of pills dispensed, indications, dose, and frequency. All other patient data, including symptoms, diagnostic testing results, and inpatient antibiotic treatment will be obtained via HMS medical record review as described previously [4, 7, 37]. Thirty-day post-discharge outcomes will be obtained through HMS via a combination of medical record review and patient phone calls. In HMS, all patients who survive to hospital discharge have 30-day outcomes obtained via medical record review. In addition, patients eligible for a discharge phone call (i.e., those not discharged to hospice, prison, or a skilled care facility or those not known to have died or been rehospitalized) are called 30 days after hospitalization (3 attempts made) to obtain information on antibiotic-related adverse effects and any additional 30-day outcome data that might be missing from the medical record. HMS has collected data on mortality, readmission, need for additional care (i.e., urgent/emergent visit), and antibiotic-associated adverse events, via this method since 2017 [7, 11, 61].

### Statistical analysis

#### Sample size

Over the study period, we anticipate including ~ 8000 patients in the intervention arm and ~ 8000 patients in the “usual stewardship” control arm (~ 400 patients/hospital/year). We estimated sample size and power based on the number of clusters (20 per arm) and using previously published data on antibiotic overuse at discharge, including in HMS hospitals [11, 35, 62], which found (a) baseline 2.2 days of antibiotic overuse at discharge per patient (or 4.4 days per patient with overuse); (b) an intraclass correlation coefficient (ICC) for our primary outcome (days of antibiotic overuse at discharge) of 0.03 to 0.035; and (c) the average number of patients included per hospital per year (400). Estimates of effect size were determined based on the minimal clinically important difference (MCID) needed to improve distal public health outcomes (20%) [63–66] and plausible intervention effect size (24.8–40%) [15–17, 19, 67]. Given a high number of zeros (i.e., many patients with no antibiotic overuse at discharge), we used a negative binomial regression to estimate power. Under these conditions, a baseline-adjusted cluster randomized trial with 40 hospitals would have 80 to > 90% power (depending on ICC estimate) to detect the MCID (a 20% absolute difference in days of antibiotic overuse at discharge) and > 90% power to detect the minimum plausible effect size (a 24.8% difference).

#### Analysis of primary outcome

Our primary intention-to-treat analysis will compare the average baseline-adjusted days of antibiotic overuse at discharge in the intervention arm vs. the usual stewardship control arm during the 12 months of study intervention. We will compare antibiotic overuse at discharge between treatment groups using a generalized linear mixed-effects model (or generalized estimating equation analysis if the mixed-effects model is numerically intractable) accounting for correlation of patients within hospitals and adjusting for baseline antibiotic overuse at discharge, as well as patient covariates that were included in the covariate-constrained randomization procedure. To account for the over-abundance of zeros in the data (i.e., no days of antibiotic overuse at discharge), we will use a negative binomial or zero-inflated Poisson outcome model [68]. We will additionally control for time since the study began in months and as a secondary analysis we will examine a treatment group interaction with time. We plan to estimate treatment effects within the following a priori subgroups: baseline discharge antibiotic overuse tertiles, social needs of population being served by the hospital (i.e., rurality, minority serving safety net, or neither), condition (i.e., CAP, UTI), and type of antibiotic overuse at discharge (i.e., unnecessary, excess duration, suboptimal fluoroquinolone). Exploratory analyses will determine the trajectory of the intervention effect (i.e., slope change, differences at 3, 6, 12, 18 months [sustainability] after intervention) and effect of different stewardship intervention combinations. We will conduct sensitivity analyses (a) dropping control hospitals that begin a Tier 3 intervention and (b) dropping intervention hospitals that fail to implement their selected ROAD Home interventions.

#### Analysis of secondary outcomes

We will repeat the above analysis for clinical patient outcomes including the composite 30-day outcome and composite 30-day antibiotic-associated adverse events. Since our secondary outcomes are binary, we will use Bernoulli outcome models with a log link to express the estimated treatment effects as ratios of the risks of discharge antibiotic overuse, as is generally preferred for clinical trials [69]. The clinical patient outcomes balancing metric will be analyzed in a non-inferiority testing framework within our generalized linear mixed-effects model, testing whether 30-day patient outcomes are not worse in the intervention arm relative to the control arm. We expect these adverse clinical outcomes to be rare, and plan to use a relative non-inferiority margin of 50% to demonstrate non-inferiority of the intervention arm to the control arm. For our secondary outcome of 30-day antibiotic adverse events, we will conduct a conventional test of differences using a 2-sided test and a 0.05 significance level.

### Mixed-methods concurrent process evaluation

To identify contextual factors influencing the implementation of the ROAD Home strategy, and to understand how tailoring works to promote or impede the uptake of evidence, we will conduct a concurrent mixed methods process evaluation [70]. Qualitative and quantitative data (observations, hospital surveys, stakeholder interviews, document analysis) will be gathered during the pre-intervention, intervention, and postintervention periods across all 20 intervention hospitals. Our Implementation Research Logic Model (Fig. 3), informed by the updated Consolidated Framework for Implementation Research (CFIR) and iPARIHS, will be used to organize our process evaluation [71].

#### Implementation outcomes

We hypothesize that tailoring will work to improve discharge antibiotic use by supporting hospitals in selecting stewardship interventions that are well-matched to the context of their inner setting. The constructs within CFIR’s inner setting domain are most relevant to understanding how well stewardship interventions “fit” with context. We will examine structural characteristics (information technology infrastructure and work infrastructure), relational connections between stewardship and prescribers, internal communications, and degree of organizational learning-centeredness [71]. Relevant inner setting constructs include tension for change, compatibility, relative priority, available resources, and access to knowledge and information. We will examine how the ROAD Home strategy interacts with these determinants to influence four implementation outcomes: acceptability, feasibility, fidelity, and sustainment (Table 2).

**Table 2:** Implementation outcomes measured in the trial

**Outcome** Definition	**Role in analysis**	**Referent**	**Data source and measurement**	**Timing and frequency**	**Unit of analysis**
					**Unit of observation**
**Acceptability** The perception among implementation stakeholders that a given treatment, service, practice, or innovation is agreeable, palatable, or satisfactory.	Independent variable	Selected stewardship interventions ROAD Home Strategy	Survey – acceptability of intervention measure; qualitative interviews	Post-intervention Once	Organization
					Individual implementation leads
**Feasibility** The extent to which a new treatment, or innovation, can be successfully used or carried out within a given agency or setting.	Independent variable	Selected stewardship interventions	Survey – feasibility of intervention measure; qualitative interviews	Post-intervention Once	Unit of analysis: organization Unit of observation: individual implementation leads
**Fidelity** The degree to which an intervention was implemented as it was prescribed in the original protocol or as it was intended by the program developers.	Dependent variable	Selected stewardship interventions ROAD Home Strategy	Observations; document analysis; qualitative interviews	Pre-intervention intervention post-intervention Ongoing	Unit of analysis: organization Unit of observation: individual implementation leads
**Sustainment** The extent to which a newly implemented treatment is maintained or institutionalized within a service setting’s ongoing, stable operations.	Dependent variable	Selected stewardship interventions	Survey: qualitative interviews; medical record review	Post-intervention Twice – end of intervention (survey, interview, medical record review) and 12 months post-intervention (survey, record review)	Unit of analysis: organization Unit of observation: individual implementation leads

#### Data collection

##### Observations

We will take detailed observational notes during all meetings between study investigators and intervention hospitals. This includes the virtual meeting we will have with each hospital to review their needs assessment, present the tailored suite, and support them in selecting 3 point’s worth of interventions. We will observe all ROAD Home intervention hospital meetings at the triannual HMS in-person meeting. Trained study staff will use an observation template informed by CFIR to take notes on the discussions and interactions that occur during the meeting. Observations will be used to ascertain fidelity to the ROAD Home strategy (e.g., do hospitals attend ROAD Home meetings, do hospitals turn in their implementation blueprint) and fidelity to the selected antibiotic stewardship interventions (e.g., do hospitals abandon the intervention(s) they initially selected, do hospitals modify the planned intervention(s), do hospitals only partially implement an intervention).

##### Survey

We will conduct two types of surveys—one to examine hospital characteristics, needs, stewardship interventions, feasibility, and acceptability and the second to examine fidelity to the implementation blueprint. First, HMS requires hospitals to respond to biannual surveys as a condition of participation [72]. Hospital characteristics that will inform the baseline assessment, including ongoing existing discharge antibiotic stewardship interventions, hospital priorities, and resources, will be obtained via the biannual HMS survey. At the end of the intervention period, we will add a series of items to the HMS survey to ascertain feasibility and acceptability of the ROAD Home strategy and the selected ROAD Home stewardship interventions. Second, we will administer a series of surveys to implementation lead(s) at months 3, 6, and 9 of the intervention period to monitor fidelity to the implementation blueprint.

##### Semi-structured interviews

In the 4 months, following the completion of the intervention period, we will conduct virtual semi-structured interviews with each intervention hospital’s implementation lead(s) and any other key stakeholders identified during the implementation process. The purpose of the interviews is to identify barriers and facilitators to implementation and assess acceptability, feasibility, fidelity, and sustainment of the discharge stewardship interventions selected. We will also ask questions about their experiences with the tailoring process. Interviews will be conducted by a trained member of the investigative team using a guide informed by CFIR with additional questions tailored to the hospital based on information gathered during observations.

##### Document analysis

We will ask intervention hospitals to share documents, tools, or guidelines they created to support implementation of selected stewardship interventions at their hospital. This can include copies of new guidelines, screenshots of clinical decision support, educational materials, and/or organizational messaging. Valuable information about implementation, including fidelity and adaptation to context, are encompassed in objects that organizations create to support the integration of an intervention into routine use [73–75].

#### Data analysis

##### Qualitative analysis

Interview transcripts, notes, and hospital artifacts will be uploaded to QSR NVivo software [76]. The codebook will be created to include a priori codes derived from CFIR, iPARHIS, and the Proctor et al. [77] taxonomy of implementation outcomes. We will augment the codebook with concepts that arise during data collection and after a period of familiarization with the data [78–80]. A sample of documents will be double-coded and inter-coder reliability assessed with discrepancies resolved by consensus. After coding, we will use framework matrices to examine variation across respondents, performance, and organizational context.

##### Quantitative analysis

Descriptive statistics (means, standard deviations, frequencies, percentages) will be used to summarize fixed‐response survey items. *T*-tests will be used for comparisons. For categorical items, responses will be examined as frequencies and comparisons will be made with the chi-square or fisher-exact test, as appropriate. For all analyses, *p* < 0.05 will be considered significant. Survey responses will also be compared by hospital characteristics.

## Discussion

The ROAD Home trial is a two-arm parallel cluster RCT of a tailored, multicomponent implementation strategy to improve the quality and safety of discharge antibiotic prescribing. It includes external facilitation to support diverse hospitals in selecting and implementing evidence-based antibiotic stewardship interventions based on local context. This will be a large-scale, multi-site study testing the impact of implementation strategies to improve uptake of stewardship across hospitals with varied implementation capacity. Too often, antibiotic stewardship research has applied a “one-size-fits-all” approach to interventions, leading to a widening implementation gap across diverse contexts—where antibiotic prescribing at academic medical centers (which tend to be heavily resourced) improves while antibiotic prescribing at critical access and community hospitals remains stagnant [45, 47, 81–83].

The ROAD Home trial is informed by the idea that achieving equity in antibiotic stewardship requires that all patients, wherever they receive care, can access the health benefits produced by stewardship, such as reductions in antibiotic-associated adverse events, optimized therapy to treat infection, and decreased transmission of antibiotic-resistant bacteria. Through inclusion of diverse hospital contexts (e.g., rural, urban, community), which are reflective of the places many vulnerable, minoritized, and marginalized patients receive care, the ROAD Home trial will generate knowledge that explicitly advances the integration of health equity into antibiotic stewardship. ROAD Home will do so by targeting the organization’s inner setting. The inner setting has been overlooked in the field’s recent increased focus on equity which has instead centered on interactions between patients and clinicians or social determinants of health in the outer setting that increase an individual’s risk of acquiring an antibiotic resistant infection [84].

In addition to specifying determinants, mechanisms, and outcomes that clarify “how” to improve antibiotic use through tailoring, there are several additional strengths of this study. First, it represents a departure from the status quo given its rigorous study design as a multi-center randomized, controlled trial that includes hospitals with a range of resources and capacities for stewardship, allowing us to closely examine the impact of hospital context on implementation. Second, we have carefully selected our implementation strategies based on known barriers and facilitators to integrating stewardship into resource-constrained settings. While these barriers have been widely identified, there is little research systematically examining strategies to overcome them. Our trial will generate knowledge that explicitly addresses the implementation gap in stewardship science more broadly, beyond our specific target of antibiotic prescribing at hospital discharge. Third, we will evaluate an innovative primary outcome which combines three types of antibiotic overuse (unnecessary, excess duration, avoidable fluoroquinolones) into a single guideline-based validated metric which will be assessed for ~ 8000 intervention patients.

The study also has limitations. It is possible that hospitals will drop out of the trial, and that hospitals with the most obstacles to improvement (whom we are trying to reach) may disproportionately drop out. We will include hospitals that drop out in an intention-to-treat analysis. Even if hospitals stop participating in the trial, it is unlikely that they will drop out of HMS (none has left the consortium in the last 2 years), which allows us to include their data. A second limitation is that we are gathering data on implementation outcomes reflective of the organization by only a small number of individuals (e.g., 1–2 implementation leads). We believe that this approach is justified because the target of action of our implementation strategies are those individuals tasked with leading antibiotic stewardship interventions in hospitals. These individuals play a critical role in changing prescriber behavior by modifying organizational-level systems or through direct interaction [85–87]. They can be thought of as “agents of implementation” [88]. Conducting this trial in HMS somewhat limits our generalizability in that the structure of the collaborative introduces incentives for hospitals to participate and focus on stewardship through pay for performance (P4P) antibiotic use measures, which do not exist for most hospitals in the US. Future work will evaluate the effect of the ROAD Home strategy outside of a pre-existing quality collaborative. Finally, although we believe it is unlikely, hospitals in our control group may implement interventions to improve discharge prescribing to achieve P4P, which may impact our ability to detect a difference. We will test for this in a sensitivity analysis but it may reduce our overall power to detect difference in outcomes.

Improving discharge antibiotic prescribing across hospitals with varied needs and resources requires tailoring of interventions to local context. The ROAD Home trial will assess whether a multi-component implementation strategy combining tailoring and external facilitation leads to reduction in antibiotic overuse at hospital discharge. Knowledge gained during this study could inform future efforts to implement stewardship and patient safety interventions in diverse hospital contexts to promote equity in access to the benefits of quality improvement initiatives.

### Supplementary Information

**Supplementary material 1.**

**Supplementary material 2.**

## Data Availability

As per existing data use agreements within the Michigan Hospital Medicine Safety Consortium, we are not able to share any individual patient data or any identified hospital level data. Aggregated patient and hospital level data will be shared via publication. Additional requests for data access can be made to the corresponding authors and will be reviewed by HMS’ Data, Design, and Publications committee.

## Abbreviations

ASB: Asymptomatic bacteriuria
ASP: Antibiotic stewardship program
BCBSM: Blue Cross Blue Shield of Michigan
CAP: Community-acquired pneumonia
CDI: *Clostridioides* *difficile* Infection
CFIR: Consolidated Framework for Implementation Research
HMS: Michigan Hospital Medicine Safety Consortium
ICC: Intraclass correlation coefficient
ID: Infectious diseases
iPARHIS: Integrated-Promoting Action on Research Implementation in Health Services
MCID: Minimal clinically important differences
P4P: Pay for performance
ROAD Home: Reducing Overuse of Antibiotics at Discharge
RUCC: Rural-urban continuum code
US: United States
UTI: Urinary tract infection

## Footnotes

1 Evidence-based antibiotic stewardship interventions often promote de-implementation since most of the evidence-practice gaps in antibiotic prescribing demonstrate that patients receive antibiotics inappropriately (e.g., antibiotics prescribed for too long of a duration, an inappropriately broad spectrum drug is used when a narrow spectrum drug would be just as effective with fewer side effects) [34]. This is the case when it comes to the use of antibiotics at hospital discharge, where patients often receive antibiotics they do not need, for too long of a duration with a suboptimal drug [4, 5, 8]. In this trial, we aim to reduce antibiotic overuse at the time of hospital discharge, which is an example of de-implementation. However, we are evaluating the impact of a multicomponent strategy to support hospital-based stakeholders in *implementing* evidence-based stewardship interventions to reduce (de-implement) antibiotic overuse at hospital discharge. Because the action target of the multicomponent strategy we are testing is antibiotic stewardship stakeholders who we want to start doing something (vs. stop doing something), we refer to the phenomenon we are focusing on in this trial as implementation and not de-implementation.

## References

[1] Tamma PD, Avdic E, Li DX, Dzintars K, Cosgrove SE (2017). Association of adverse events with antibiotic use in hospitalized patients. JAMA Intern Med.

[2] Akpoji UC, Wilson BM, Briggs JM (2022). Antibiotic exposure and acquisition of antibiotic-resistant gram-negative bacteria among outpatients at a US Veterans Affairs medical center. Antimicrob Steward Healthc Epidemiol.

[3] Curran J, Lo J, Leung V (2022). Estimating daily antibiotic harms: an umbrella review with individual study meta-analysis. Clin Microbiol Infect.

[4] Vaughn VM, Hersh AL, Spivak ES (2022). Antibiotic overuse and stewardship at hospital discharge: the reducing overuse of antibiotics at discharge home framework. Clin Infect Dis.

[5] Yogo N, Haas MK, Knepper BC, Burman WJ, Mehler PS, Jenkins TC (2015). Antibiotic prescribing at the transition from hospitalization to discharge: a target for antibiotic stewardship. Infect Control Hosp Epidemiol.

[6] Dyer AP, Dodds Ashley E, Anderson DJ (2019). Total duration of antimicrobial therapy resulting from inpatient hospitalization. Infect Control Hosp Epidemiol.

[7] Vaughn VM, Flanders SA, Snyder A (2019). Excess antibiotic treatment duration and adverse events in patients hospitalized with pneumonia: a multihospital cohort study. Ann Intern Med.

[8] Yi SH, Hatfield KM, Baggs J (2018). Duration of antibiotic use among adults with uncomplicated community-acquired pneumonia requiring hospitalization in the United States. Clin Infect Dis.

[9] Feller J, Lund BC, Perencevich EN (2020). Post-discharge oral antimicrobial use among hospitalized patients across an integrated national healthcare network. Clin Microbiol Infect.

[10] Fridkin S, Baggs J, Fagan R (2014). Vital signs: improving antibiotic use among hospitalized patients. MMWR Morb Mortal Wkly Rep.

[11] Vaughn VM, Gandhi TN, Chopra V (2021). Antibiotic overuse after hospital discharge: a multi-hospital cohort study. Clin Infect Dis.

[12] Weber BR, Noble BN, Bearden DT (2019). Antibiotic prescribing upon discharge from the hospital to long-term care facilities: implications for antimicrobial stewardship requirements in post-acute settings. Infect Control Hosp Epidemiol.

[13] Gontjes KJ, Gibson KE, Lansing BJ (2022). Association of exposure to high-risk antibiotics in acute care hospitals with multidrug-resistant organism burden in nursing homes. JAMA Netw Open.

[14] Low M, Neuberger A, Hooton TM (2019). Association between urinary community-acquired fluoroquinolone-resistant Escherichia coli and neighbourhood antibiotic consumption: a population-based case-control study. Lancet Infect Dis.

[15] Daniels LM, Weber DJ (2021). Interventions to improve antibiotic prescribing at hospital discharge: a systematic review. Infect Control Hosp Epidemiol.

[16] Giesler DL, Krein S, Brancaccio A (2022). Reducing overuse of antibiotics at discharge home: a single-center mixed methods pilot study. Am J Infect Control.

[17] Yogo N, Shihadeh K, Young H (2017). Intervention to reduce broad-spectrum antibiotics and treatment durations prescribed at the time of hospital discharge: a novel stewardship approach. Infect Control Hosp Epidemiol.

[18] Schuler CL, Courter JD, Conneely SE (2016). Decreasing duration of antibiotic prescribing for uncomplicated skin and soft tissue infections. Pediatrics.

[19] Mercuro NJ, Medler CJ, Kenney RM (2022). Pharmacist-driven transitions of care practice model for prescribing oral antimicrobials at hospital discharge. JAMA Netw Open.

[20] Saleh J, El Nekidy WS, El Lababidi R (2022). Assessment of antibiotic appropriateness at discharge: experience from a quaternary care hospital setting. JAC Antimicrob Resist.

[21] McGregor JC, Fitzpatrick MA, Suda KJ (2021). Expanding antimicrobial stewardship through quality improvement. JAMA Netw Open.

[22] Vaughn VM, Krein SL, Hersh A, Buckel WR, White AT, Horowitz J, et al. Excellence in antibiotic stewardship: a mixed methods study comparing high, medium, and low performing hospitals. Clin Infect Dis. 2023:ciad743. 10.1093/cid/ciad743. PMID: 38059532. 10.1093/cid/ciad743 PMC11153329 38059532

[23] Szymczak JE, Fiks AG, Craig S, Mendez DD, Ray KN (2023). Access to what for whom? How care delivery innovations impact health equity. J Gen Intern Med.

[24] Brownson RC, Kumanyika SK, Kreuter MW, Haire-Joshu D (2021). Implementation science should give higher priority to health equity. Implement Sci.

[25] Kwan BM, Brownson RC, Glasgow RE, Morrato EH, Luke DA (2022). Designing for dissemination and sustainability to promote equitable impacts on health. Annu Rev Public Health.

[26] Baumann AA, Cabassa LJ (2020). Reframing implementation science to address inequities in healthcare delivery. BMC Health Serv Res.

[27] Brownson RC, Jacobs JA, Tabak RG, Hoehner CM, Stamatakis KA (2013). Designing for dissemination among public health researchers: findings from a national survey in the United States. Am J Public Health.

[28] Patel PK (2020). One size doesn’t fit all-stewardship interventions need to be tailored in large healthcare systems. Clin Infect Dis.

[29] Szymczak JE (2019). Are surgeons different? The case for bespoke antimicrobial stewardship. Clin Infect Dis.

[30] Sikkens JJ, van Agtmael MA, Peters EJG (2017). Behavioral approach to appropriate antimicrobial prescribing in hospitals: the Dutch Unique Method for Antimicrobial Stewardship (DUMAS) participatory intervention study. JAMA Intern Med.

[31] Duncan EM, Charani E, Clarkson JE (2020). A behavioural approach to specifying interventions: what insights can be gained for the reporting and implementation of interventions to reduce antibiotic use in hospitals?. J Antimicrob Chemother.

[32] McHugh SM, Riordan F, Curran GM (2022). Conceptual tensions and practical trade-offs in tailoring implementation interventions. Front Health Serv.

[33] Tamma PD, Miller MA, Cosgrove SE (2019). Rethinking how antibiotics are prescribed: incorporating the 4 moments of antibiotic decision making into clinical practice. JAMA.

[34] Livorsi DJ, Drainoni ML, Reisinger HS (2022). Leveraging implementation science to advance antibiotic stewardship practice and research. Infect Control Hosp Epidemiol.

[35] Vaughn VM, Ratz D, Greene MT (2022). Antibiotic stewardship strategies and their association with antibiotic overuse after hospital discharge: an analysis of the reducing overuse of antibiotics at discharge (road) home framework. Clin Infect Dis.

[36] Vaughn VM, Gandhi TN, Hofer TP (2022). A statewide collaborative quality initiative to improve antibiotic duration and outcomes in patients hospitalized with uncomplicated community-acquired pneumonia. Clin Infect Dis.

[37] Vaughn VM, Gupta A, Petty LA (2023). A statewide quality initiative to reduce unnecessary antibiotic treatment of asymptomatic bacteriuria. JAMA Intern Med.

[38] Jake-Schoffman DE, Brown SD, Baiocchi M (2021). Methods-motivational interviewing approach for enhanced retention and attendance. Am J Prev Med.

[39] Goldberg JH, Kiernan M (2005). Innovative techniques to address retention in a behavioral weight-loss trial. Health Educ Res.

[40] Li F, Lokhnygina Y, Murray DM, Heagerty PJ, DeLong ER (2016). An evaluation of constrained randomization for the design and analysis of group-randomized trials. Stat Med.

[41] USDA. Rural-Urban continuum codes. Economic Research Service, U.S. Department of Agriculture; 2022. https://www.ers.usda.gov/data-products/rural-urban-continuum-codes.aspx. Accessed 8 Jan 2022.

[42] 2022 national healthcare quality and disparities report. Rockville: Agency for Healthcare Research and Quality; 2022. https://www.ahrq.gov/sites/default/files/wysiwyg/research/findings/nhqrdr/2022qdr.pdf. Accessed 18 Feb 2024. AHRQ Pub. No. 22(23)-00302022. 36475568

[43] Vaughn VM, Gandhi T, Conlon A, Chopra V, Malani AN, Flanders SA (2019). The association of antibiotic stewardship with fluoroquinolone prescribing in michigan hospitals: a multi-hospital cohort study. Clin Infect Dis.

[44] Vaughn VM, Seelye SM, Wang XQ, Wiitala WL, Rubin MA, Prescott HC (2020). Inpatient and discharge fluoroquinolone prescribing in veterans affairs hospitals between 2014 and 2017. Open Forum Infect Dis.

[45] Advani SD, Sickbert-Bennett E, Moehring R (2023). The disproportionate impact of coronavirus disease 2019 (COVID-19) pandemic on healthcare-associated infections in community hospitals: need for expanding the infectious disease workforce. Clin Infect Dis.

[46] Livorsi DJ, Reisinger HS, Stenehjem E (2019). Adapting antibiotic stewardship to the community hospital. JAMA Netw Open.

[47] Livorsi DJ, Stewart Steffensmeier KR, Perencevich EN, Goetz MB, Schacht Reisinger H (2022). Antibiotic stewardship implementation at hospitals without on-site infectious disease specialists: a qualitative study. Infect Control Hosp Epidemiol.

[48] Krockow EM, Colman AM, Chattoe-Brown E (2019). Balancing the risks to individual and society: a systematic review and synthesis of qualitative research on antibiotic prescribing behaviour in hospitals. J Hosp Infect.

[49] Lorencatto F, Charani E, Sevdalis N, Tarrant C, Davey P (2018). Driving sustainable change in antimicrobial prescribing practice: how can social and behavioural sciences help?. J Antimicrob Chemother.

[50] Huang J, Kassamali Escobar Z, Bouchard TS (2021). Finding the path of least resistance: locally adapting the MITIGATE toolkit in emergency departments and urgent care centers. Infect Control Hosp Epidemiol.

[51] Yadav K, Stahmer A, Mistry RD, May L (2020). An implementation science approach to antibiotic stewardship in emergency departments and urgent care centers. Acad Emerg Med.

[52] Nace DA, Hanlon JT, Crnich CJ (2020). A multifaceted antimicrobial stewardship program for the treatment of uncomplicated cystitis in nursing home residents. JAMA Intern Med.

[53] van Buul LW, Sikkens JJ, van Agtmael MA, Kramer MH, van der Steen JT, Hertogh CM (2014). Participatory action research in antimicrobial stewardship: a novel approach to improving antimicrobial prescribing in hospitals and long-term care facilities. J Antimicrob Chemother.

[54] Waltz TJ, Powell BJ, Matthieu MM (2015). Use of concept mapping to characterize relationships among implementation strategies and assess their feasibility and importance: results from the Expert Recommendations for Implementing Change (ERIC) study. Implement Sci.

[55] Harvey G, Kitson A (2016). PARIHS revisited: from heuristic to integrated framework for the successful implementation of knowledge into practice. Implement Sci.

[56] Harvey G, Kitson A. Implementing evidence-based practice in healthcare: a facilitation guide. 1st ed. Routledge; 2015. 10.4324/9780203557334.

[57] Proctor EK, Powell BJ, McMillen JC (2013). Implementation strategies: recommendations for specifying and reporting. Implement Sci.

[58] Smith JD, Li DH, Rafferty MR (2020). The Implementation Research Logic Model: a method for planning, executing, reporting, and synthesizing implementation projects. Implement Sci.

[59] Weiner BJ, Lewis CC, Sherr K, et al. Practical implementation science. 1st ed. In: Moving evidence into action. New York: Springer Publishing Company; 2022. 10.1891/9780826186935.

[60] Uranga A, Artaraz A, Bilbao A (2020). Impact of reducing the duration of antibiotic treatment on the long-term prognosis of community acquired pneumonia. BMC Pulm Med.

[61] Petty LA, Vaughn VM, Flanders SA (2019). Risk factors and outcomes associated with treatment of asymptomatic bacteriuria in hospitalized patients. JAMA Intern Med.

[62] Merlo J, Yang M, Chaix B, Lynch J, Råstam L (2005). A brief conceptual tutorial on multilevel analysis in social epidemiology: investigating contextual phenomena in different groups of people. J Epidemiol Community Health.

[63] Lee TC, Frenette C, Jayaraman D, Green L, Pilote L (2014). Antibiotic self-stewardship: trainee-led structured antibiotic time-outs to improve antimicrobial use. Ann Intern Med.

[64] MacFadden DR, Fisman DN, Hanage WP, Lipsitch M (2019). The relative impact of community and hospital antibiotic use on the selection of extended-spectrum beta-lactamase-producing Escherichia coli. Clinical Infect Dis.

[65] Timbrook TT, Hurst JM, Bosso JA (2016). Impact of an antimicrobial stewardship program on antimicrobial utilization, bacterial susceptibilities, and financial expenditures at an academic medical center. Hosp Pharm.

[66] Barnes SL, Rock C, Harris AD, Cosgrove SE, Morgan DJ, Thom KA (2017). The impact of reducing antibiotics on the transmission of multidrug-resistant organisms. Infect Control Hosp Epidemiol.

[67] Chavada R, Davey J, O’Connor L, Tong D (2018). ‘Careful goodbye at the door’: is there role for antimicrobial stewardship interventions for antimicrobial therapy prescribed on hospital discharge?. BMC Infect Dis.

[68] Yang S, Harlow LL, Puggioni G, Redding CA (2017). A comparison of different methods of zero-inflated data analysis and an application in health surveys. J Mod Appl Stat Methods.

[69] McNutt LA, Wu C, Xue X, Hafner JP (2003). Estimating the relative risk in cohort studies and clinical trials of common outcomes. Am J Epidemiol.

[70] Grant A, Treweek S, Dreischulte T, Foy R, Guthrie B (2013). Process evaluations for cluster-randomised trials of complex interventions: a proposed framework for design and reporting. Trials.

[71] Damschroder LJ, Reardon CM, Widerquist MAO, Lowery J (2022). The updated consolidated framework for implementation research based on user feedback. Implement Sci.

[72] Vaughn VM, Petty LA, Flanders SA (2020). A deeper dive into antibiotic stewardship needs: a multihospital survey. Open Forum Infect Dis.

[73] Kriznik NM, Lamé G, Dixon-Woods M (2019). Challenges in making standardisation work in healthcare: lessons from a qualitative interview study of a line-labelling policy in a UK region. BMJ Open.

[74] Woodcock T, Liberati EG, Dixon-Woods M (2021). A mixed-methods study of challenges experienced by clinical teams in measuring improvement. BMJ Qual Saf.

[75] Dixon-Woods M, Redwood S, Leslie M, Minion J, Martin GP, Coleman JJ (2013). Improving quality and safety of care using “technovigilance”: an ethnographic case study of secondary use of data from an electronic prescribing and decision support system. Milbank Q.

[76] NVivo. Version 12. 2018. https://www.qsrinternational.com/nvivo-qualitative-data-analysis-software/home. Accessed 18 Feb 2024.

[77] Proctor E, Silmere H, Raghavan R, Hovmand P, Aarons G, Bunger A (2011). Outcomes for implementation research: conceptual distinctions, measurement challenges, and research agenda. Adm Policy Ment Health.

[78] Miles M, Huberman A, Saldana J (2014). Qualitative data analysis: a methods sourcebook.

[79] Deterding NM, Waters MC (2021). Flexible coding of in-depth interviews: a twenty-first-century approach. Sociol Methods Res.

[80] Gale NK, Heath G, Cameron E, Rashid S, Redwood S (2013). Using the framework method for the analysis of qualitative data in multi-disciplinary health research. BMC Med Res Methodol.

[81] CDC. Core elements of hospital antibiotic stewardship programs. US Department of Health and Human Services. CDC. https://www.cdc.gov/antibiotic-use/healthcare/pdfs/hospital-core-elements-H.pdf. Accessed 3 Aug 2022.

[82] CDC. Antibiotic use in rural hospitals. Center for Surveillance, Epidemiology, and Laboratory Services (CSELS); 2022. https://www.cdc.gov/ruralhealth/antibiotic/index.html. Accessed 3 Aug 2022.

[83] Stenehjem E, Hyun DY, Septimus E (2017). Antibiotic stewardship in small hospitals: barriers and potential solutions. Clin Infect Dis.

[84] Woodward EN, Matthieu MM, Uchendu US, Rogal S, Kirchner JE (2019). The health equity implementation framework: proposal and preliminary study of hepatitis C virus treatment. Implement Sci.

[85] Vaughn VM, Giesler DL, Mashrah D, Brancaccio A, Sandison K, Spivak ES (2023). Pharmacist gender and physician acceptance of antibiotic stewardship recommendations: an analysis of the reducing overuse of antibiotics at discharge home intervention. Infect Control Hosp Epidemiol.

[86] Szymczak J, Muller B, Shakamuri N (2021). The impact of social role identity on communication in hospital-based antimicrobial stewardship. Antimicrob Steward Healthc Epidemiol.

[87] Szymczak JE, Elle Saine M, Chiotos K, et al. 965. Prevalence and drivers of burnout among antimicrobial stewardship personnel in the United States: a cross-sectional study. Open Forum Infect Dis. 2022;9(Supplement_2). 10.1093/ofid/ofac492.808.

[88] Wensing M, Sales A, Wilson P (2021). Implementation Science and Implementation Science Communications: a refreshed description of the journals’ scope and expectations. Implement Sci.

