# Supplementary material for: Protocol for a parallel cluster randomized trial of a participatory tailored approach to reduce overuse of antibiotics at hospital discharge: the ROAD home trial
Source: Implement Sci. 2024 Mar 4;19:23. doi: 10.1186/s13012-024-01348-w (PMC10910678; doi:10.1186/s13012-024-01348-w)
Supplement: Supplementary file 1 — Supplementary material 1. [file 13012_2024_1348_MOESM1_ESM.pdf]

Additional File 1

Table. ROAD Home Implementation Strategies

ERIC[1] Domain	Use Evaluative and Iterative Strategies		Adapt and Tailor to Context	Provide Interactive Assistance	Develop Stakeholder Interrelationships
Individual Strategies from ERIC	-Conduct a local needs assessment<br>-Assess for readiness and identify barriers and facilitators	-Develop a formal implementation blueprint	-Tailor strategies<br>-Promote adaptability	-Facilitation	-Identify and prepare champions
			Specification		
Actor	ROAD Home research team who are experts in antibiotic stewardship at hospital discharge.	ROAD Home Research Team	ROAD Home Research Team; Inner Setting Implementation Leads	ROAD Home Research Team	ROAD Home Research Team
Action	Uses structured surveys to ascertain contextual determinants likely to influence implementation; facilitate discussion with implementation leads about barriers and facilitators to discharge stewardship, develops and shares a formal implementation blueprint.	Use an implementation blueprint to be filled out by intervention hospitals to specify their plans for implementation in advance.	Use information about local needs, readiness, determinants to create customized suite of weighted stewardship strategies. Inner Setting Implementation Leads have free choice of what strategies to implement if they total 3 points.	Provide support to Inner Setting Implementation Leads through prescribing performance data analysis, provision of customized suite, provision of implementation blueprint; provision of adaptable materials (e.g. education, literature reviews, expert stewardship guidance); ongoing availability for challenges that arise	Use a bundle of strategies (implement-tation blueprint, needs assessment, assessment of readiness) to prepare Inner Setting Implementation Leads to implement stewardship strategies in their hospital to improve discharge antibiotic prescribing.
Target(s) of Action	Inner setting implementation leads – antimicrobial stewardship stakeholders (physician, pharmacist, administrative leader).	Inner setting implementation leads	Inner setting implementation leads; Clinicians (indirectly)	Inner setting implementation leads	Inner setting implementation leads

Temporality	18 months prior to local site implementation of selected stewardship strategies	7 months prior to local site implementation	12 months prior to local site implementation	18 months prior to local site implementation;<br>12 month intervention period	18 months prior to local site implementation
Dose	-1 survey with implementation leads to determine local needs, readiness, and identify potential barriers and facilitators<br>-Medical record review of discharge prescribing performance to inform stewardship targets	-3 points of engagement with the blueprint: pre-intervention and 2 times during intervention – blueprint updates	-1-2-hour meeting between ROAD Home research team and implementation leads to discuss customized suite of stewardship strategies and provide instructions on how to fill out implementation blueprint	-1-2-hour meeting between ROAD Home research team and implementation leads to discuss and select stewardship strategies to implement and provide instructions on how to fill out implementation blueprint<br><br>-3 1 hour in person meeting at HMS collaborative<br>-Ongoing feedback on implementation blueprints	-1-2-hour meeting between ROAD Home research team and implementation leads to discuss and select stewardship strategies to implement and provide instructions on how to fill out implementation blueprint<br><br>-Email feedback at one point in time on initial implementation blueprint
Implementation Outcome Affected	Acceptability<br>Feasibility<br>Fidelity	Feasibility<br>Fidelity	Acceptability<br>Feasibility<br>Fidelity	Fidelity<br>Sustainment	Acceptability<br>Fidelity<br>Sustainment
Justification	Assessing a hospital's needs, resources, and priorities related to discharge stewardship will raise awareness of problems that can inform selection of strategies that lead to increased coherence between hospital context and stewardship strategies, leading to an increase in acceptability, feasibility, fidelity to the planned approach, and sustainment.		Tailoring discharge stewardship strategies to each hospital's context will increase compatibility, increase alignment with existing organizational priorities (leading to increased acceptability), and decrease the impact of resource constraints on feasibility and fidelity.	Providing external facilitation through a QI collaborative that centralizes technical assistance and offers an opportunity to participate in a discharge stewardship intervention will increase motivation of hospitals to focus on this issue and decrease barriers to activate a	Providing external facilitation to assist inner setting implementation leads to prepare for implementing discharge stewardship will increase their self-efficacy (capability) to lead change and save them time (opportunity), leading to increase in acceptability,

			project in this area by providing stewardship tools that are accessible, well-designed and come from a source that is credible, leading to an increase in acceptability, fidelity, and sustainment.	fidelity, and sustainment.

[1]Expert Recommendations for Implementing Change (ERIC)
