# Supplementary material for: Protocol for a parallel cluster randomized trial of a participatory tailored approach to reduce overuse of antibiotics at hospital discharge: the ROAD home trial
Source: Implement Sci. 2024 Mar 4;19:23. doi: 10.1186/s13012-024-01348-w (PMC10910678; doi:10.1186/s13012-024-01348-w)
Supplement: Supplementary file 2 — Supplementary material 2. [file 13012_2024_1348_MOESM2_ESM.pdf]

Additional File 2

Figure. Example Implementation Blueprint

Self-Directed ROAD Home Implementation Blueprint

Please fill out completely and return to ROAD Home Study Team.

<u>Goals – ROAD Home Strategies (3 points)</u>

1. Updated Guidelines – Discharge Specific Recommendations (Tier 1, 1 point)
2. Audit & Feedback - Add Proactive Discussion of Discharge Antibiotics (Tier 2, 2 points)

Dates of Intervention Period: [DATES SELECTED BY HOSPITAL AND STUDY TEAM] *Total Duration of Intervention:* 12 months

**Goal and Deadline**	**Action Step**	**Responsible Individual(s)**	**Planned Timeline**	**Anticipated Barriers**	**Strategies to Address Barriers**
Update Guidelines to Include Discharge-Specific Recommendations <u>Update Guidelines by [DATE]</u>	Gain consensus on oral de-escalation guidelines	ASP Lead; Pharmacy Stakeholders; Formulary Committee	Complete by [DATE]	-Disagreement about antibiotic selections to highlight in guidelines	-Engage guideline stakeholders early<br>-ROAD Home team to provide updated evidence and materials
	Modify text in existing guidelines to state "recommended duration 3-5 days (including discharge prescription)"	ASP Lead; Pharmacy Stakeholders; Guideline Committee; IT	Complete by [DATE]	-Prioritization of this guideline change in a timely fashion	-Engage guideline gatekeepers early<br>-Align with other planned guideline changes
	Modify text in existing guidelines to include new oral de-escalation guidelines	ASP Lead; Pharmacy Stakeholders; Guideline Committee; IT	Complete by [DATE]	-Late disagreement with suggested changes	-Ensure all stakeholder engaged during consensus stage
	Circulate informational message letting key stakeholders know about changes made to guidelines	Site Coordinator	Ongoing Monthly, [DATES]	-Email overload<br>-Reaching part-time or rotating staff	-Alternatives to email – QR code at meetings or on signs<br>-Have messages come from leadership with supportive note
Audit and Provide Feedback – Add Proactive Discussion of Discharge Antibiotics <u>Start Conversations by [DATE]</u>	Determine messages about discharge antibiotics that can be integrated into already-existing daily audit and feedback	ASP Lead; ASP staff; Floor Pharmacist	Complete by [DATE]	-Additional effort needed from ASP<br>-Some reluctance to add work	-Find ways to incorporate into existing ASP workflows<br>-Engage floor pharmacists as champions
	Set a goal for number of proactive discussions to have per week	ASP lead; Physician Lead; other ASP stakeholders	Complete by [DATE]	-Group may disagree with how many recommendations to target	-Select an initial goal, modify up or down based on experience
